# Reflective, formative, hybrid, and composite measurement models in social science research: a diagnostic construct–measurement alignment framework

**DOI:** 10.3389/frma.2026.1870385

**Published:** 2026-09-24

**Authors:** Taisith Kruasom

**Affiliations:** Faculty of Management Science, Ubon Ratchathani University, Ubon Ratchathani, Thailand

**Keywords:** construct validity, formative measurement, hybrid constructs, measurement model specification, reflective measurement, research transparency, structural equation modeling

## Abstract

Measurement-model specification remains a persistent source of conceptual and statistical error in social science research. Existing guidance distinguishes reflective, causal-indicator/formative, hybrid, higher-order, and composite models, yet applied studies often select among them through disciplinary convention, software defaults, or *post hoc* empirical performance. This conceptual article reframes the problem as construct–measurement alignment and develops an explanatory and diagnostic framework rather than another binary decision rule. A structured conceptual synthesis was used to compare foundational and contemporary literature across five domains: construct ontology, indicator role, formal specification, validation logic, and contextual interpretation. The analysis integrates common-factor CFA and SEM, MIMIC models, higher-order structures, and composite-based SEM, with explicit attention to identification, measurement error, and empirically equivalent models. From inconsistencies across these five domains, the framework derives a taxonomy of seven recurrent forms of misalignment: ontological–structural, indicator–construct, epistemological–statistical, conceptual–identification, error–specification, level-of-abstraction, and context–interpretation misalignment. Three worked applications demonstrate that the same construct label can warrant different model specifications when its definition, level, purpose, or indicator role changes. The framework therefore explains why measurement decisions fail, identifies where misalignment occurs, and specifies the evidence required for diagnosis and correction. It offers researchers, reviewers, and editors a transparent basis for model justification while limiting claims that statistical fit alone can establish construct meaning or causal direction.

## 1 Introduction

### 1.1 Background and methodological significance

Measurement-model specification determines how theoretical constructs are translated into observable evidence. Constructs such as trust, satisfaction, institutional capacity, digital readiness, resilience, governance quality, and sustainability are not self-interpreting variables. Their empirical meaning depends on whether indicators are treated as manifestations, causes, constitutive components, proxies, or scoring inputs. That decision affects parameter interpretation, validity evidence, treatment of error, and the meaning of structural relations estimated later in an analysis.

The reflective–formative distinction has been central to this debate, but the binary vocabulary is now insufficient. Reflective common-factor models, causal-indicator models, composites, MIMIC models, and higher-order structures answer different questions and impose different restrictions. Misclassifying these relations can alter construct meaning, bias structural estimates, and encourage the deletion of indicators for statistical reasons that conflict with the construct domain (Jarvis et al., 2003; MacKenzie et al., 2005; Aguirre-Urreta et al., 2024).

The problem is especially consequential for research metrics and analytics. Indices and latent scores are used to compare institutions, evaluate policy, rank performance, test theories, and accumulate evidence across disciplines. A measure can appear reliable or predictively useful while remaining ontologically ambiguous. Conversely, a theoretically defensible multidimensional construct may show low internal consistency because its components are intentionally non-interchangeable. Methodological transparency therefore requires more than reporting coefficient alpha, loadings, weights, or fit indices; it requires a traceable argument from construct meaning to formal specification and interpretation.

### 1.2 Problem statement

Applied research often reverses the proper order of reasoning. Researchers begin with available indicators, software routines, or familiar validation thresholds and then infer the nature of the construct from the output. This creates an epistemological inversion: empirical behavior is treated as if it defined construct ontology. Strong correlations are interpreted as proof of reflection, weak loadings as evidence that an indicator is dispensable, and favorable fit as confirmation of causal direction. None of these inferences follows without additional theoretical assumptions.

Three linked limitations motivate this article. First, much guidance remains classificatory: it lists differences between reflective and formative models without explaining how cross-domain inconsistencies generate specific forms of misspecification. Second, the literature is fragmented across common-factor SEM, causal-indicator models, composite-based SEM, MIMIC models, and higher-order structures. Third, applied decision rules often omit identification and model equivalence, even though an inadmissible or observationally equivalent specification cannot be rescued by acceptable global fit.

### 1.3 Purpose, questions, and contribution

This article develops a theory-informed diagnostic framework for construct–measurement alignment. It asks three questions: (1) Which conceptual and formal decisions must align before a measurement model is defensible? (2) What recurrent forms of misalignment can be identified, and what consequences follow from each? (3) How does the framework alter measurement decisions when the same construct label is used in different contexts?

The contribution is not the claim that reflective and formative distinctions are new. Rather, it lies in integrating five decision domains into an iterative explanatory and diagnostic framework and deriving a seven-type taxonomy of misalignment from cross-domain inconsistencies. The framework links construct ontology, indicator role, formal specification, validation logic, and contextual interpretation. It also places identification, measurement error, and equivalent models inside the decision process rather than treating them as post-estimation technicalities. This integration provides a structured basis for identifying where construct–measurement reasoning fails and what evidence is required for diagnosis and correction.

The article further distinguishes testable theoretical expectations from methodological recommendations. Theoretical expectations specify directional or conditional relationships that can be examined empirically, whereas recommendations translate the framework into design and reporting actions. This separation is necessary because a prescription such as “justify indicator roles” is not itself a testable theoretical expectation. Finally, worked applications demonstrate diagnostic utility by showing why socioeconomic status, digital capability, and service quality can warrant different specifications across purposes and levels of analysis.

## 2 Method of conceptual synthesis

### 2.1 Design and scope

The study is a structured conceptual synthesis rather than a systematic review, meta-analysis, or bibliometric study. Its objective is theory clarification and framework development. The unit of analysis is the argument linking a construct to its indicators, including the assumed direction of dependence, the status of the construct, the role of error, the formal restrictions imposed, and the validation evidence considered relevant.

The synthesis covered five bodies of literature: construct validity and indicator specification; common-factor CFA and covariance-based SEM; causal-indicator and formative models; component- and composite-based SEM; and identification, graphical representation, higher-order modeling, and equivalent models. Foundational sources were retained when they established a model family or enduring controversy, while contemporary sources were used to capture current treatments of composites, measurement error, reporting transparency, and construct comparability.

### 2.2 Analytical procedure

The synthesis proceeded in six stages. First, theoretical and methodological statements in the literature were coded according to construct ontology, indicator role, formal specification, validation logic, and contextual interpretation. Second, terminology was disambiguated, particularly the differences among latent variables, causal indicators, composites, indices, and covariates. Third, areas of convergence and disagreement were compared across model families. Fourth, recurrent specification failures were mapped to the decision domain in which the inconsistency originated. Fifth, these mappings were consolidated into a taxonomy of construct–measurement misalignment. Sixth, the framework was applied to three worked applications to examine whether it generated coherent and context-sensitive diagnostic decisions.

This procedure does not treat frequency of citation as proof of conceptual validity. Methodological currency is relevant because applied practice can lag behind updated guidance, but newer sources are not automatically superior. Sabol et al. (2023) and Sabol and Winton (2026) are used as evidence that reporting and citation practices remain connected to methodological rigor, not as substitutes for evaluating the substantive logic of each measurement model.

### 2.3 Boundaries of inference

The article does not estimate an empirical model and does not claim that the framework has already achieved predictive validity or inter-rater reliability. Its claims are conceptual: the framework identifies necessary questions, specifies recurrent inconsistencies, and derives testable theoretical expectations suitable for later simulation, coding, and comparative empirical tests. Statistical adequacy remains essential, but it is treated as conditional on a coherent construct theory and an identified formal model.

## 3 Conceptual foundations of construct–indicator relations

### 3.1 Definition, operationalization, and measurement hypothesis

A conceptual definition states what a construct means and where its domain ends. Operationalization identifies the observations or procedures used to represent that construct. A measurement specification then states a hypothesized relation between the construct and those observations. These levels should not be collapsed. A statement such as “trust causes responses to three confidence items” is a substantive measurement hypothesis, not a logical equivalence relation. Hempel's analysis of theoretical terms is relevant here because links between theoretical and observational vocabularies require bridge assumptions and empirical support; they are not true merely by definition (Hempel, 2001, p. 208–217).

The distinction is consequential for reflective, formative, and composite specifications. A reflective equation does not define a construct as the sum of its indicators; rather, it hypothesizes that a common latent variable accounts for their covariation. Likewise, a causal-indicator equation does not establish that the selected indicators exhaust the construct domain. It hypothesizes that specified observed causes generate variation in a theoretical variable, while the disturbance term represents omitted causes. A composite equation, by contrast, can algebraically define a composite score under an explicitly specified weighting rule; however, this construction rule does not by itself establish the substantive meaning or validity of the resulting construct. That meaning remains a theoretical claim requiring justification.

Accordingly, conceptual definition, operationalization, measurement hypothesis, composite construction, formal specification, and empirical evaluation should be treated as analytically distinct stages in construct–measurement reasoning. Table 1 summarizes the function of each stage, the type of claim involved, and the principal error that arises when these levels are conflated.

**Table 1:** Conceptual distinctions required before model specification.

Level	Core question	Typical product	Frequent error
Conceptual definition	What does the construct mean, and what is outside its domain?	Domain statement and boundary conditions	Using a variable label as if it were a definition
Operationalization	Which observations or procedures represent the domain?	Items, indicators, records, or scoring rules	Selecting indicators only because they are available
Measurement hypothesis	How are construct and indicators hypothesized to relate?	Reflective, causal-indicator, MIMIC, or mixed relation	Treating direction as a statistical discovery
Composite construction	How is a composite explicitly constructed from its components?	Weighting or scoring rule	Treating an algebraically constructed score as if its substantive meaning or ontology were established by the construction rule
Formal specification	Which parameters, residuals, constraints, and scales represent the hypothesis or construction rule?	Equations, path diagram, covariance implications, or explicit construction rule	Omitting error, scale, or identification restrictions
Empirical evaluation	Which observations would be inconsistent with the specification?	Fit, residual, relevance, validity, and sensitivity evidence	Using one universal validation checklist

The levels are analytically distinct. Empirical results may challenge a measurement hypothesis or the adequacy of a construction rule, but they do not independently supply a construct definition.

### 3.2 Construct ontology

Construct ontology concerns the type of entity posited by the analysis. A latent common factor is introduced to account for shared variation among effects or manifestations. A theoretical variable represented by causal indicators is influenced by specified observed causes while remaining distinct from them through an omitted-cause disturbance. A composite is an exact or model-based linear combination of components. An index is an administratively or analytically constructed score, often used for ranking or monitoring. An emergent variable is constituted by components and may acquire substantive meaning at a higher level of organization.

These categories are not interchangeable. Bollen and Bauldry (2011) distinguish causal indicators, composite indicators, and covariates because identical arrow directions can mask different interpretations. (Kline, 2023, p. 217–228) likewise separates reflective, causal–formative, composite, and mixed-model measurement. The construct label alone cannot settle the issue. “Resilience,” for example, can refer to a latent disposition, a set of resources, a constructed index, or an emergent system property.

### 3.3 Indicator roles

Indicators may serve as effects, causes, components, proxies, or scoring inputs. Effect indicators are outcomes of a common variable and are expected to share variance for theoretically relevant reasons. Causal indicators are antecedents of a theoretical variable but do not necessarily exhaust its causes. Components participate in the construction of a composite. Proxies stand in for a target that is not directly measured. Scoring inputs are combined through an explicit rule, which may be normative rather than empirically estimated.

Interchangeability and omission are useful but not infallible diagnostics. If indicators are truly effect indicators, removing one may reduce reliability while leaving the construct domain largely intact. If indicators are constitutive components, omission can redefine the construct. However, interchangeability must be evaluated conceptually, not inferred from high correlation alone. Correlation can arise from common method, shared wording, or environmental exposure; non-correlation can coexist with a coherent composite whose components are intentionally heterogeneous.

## 4 Measurement-model families and formalization

Formalization makes the implied assumptions inspectable. Path diagrams and directed graphs are not decorative additions; they expose arrow direction, residual terms, omitted relations, and levels of abstraction. The diagrams in Figure 1 use conventional SEM notation and should be read as hypothesized structures, not as proof of causal direction. (Bollen, 2026, p. 38–46) provides a detailed treatment of the correspondence between path diagrams and equations.

**Figure 1:**
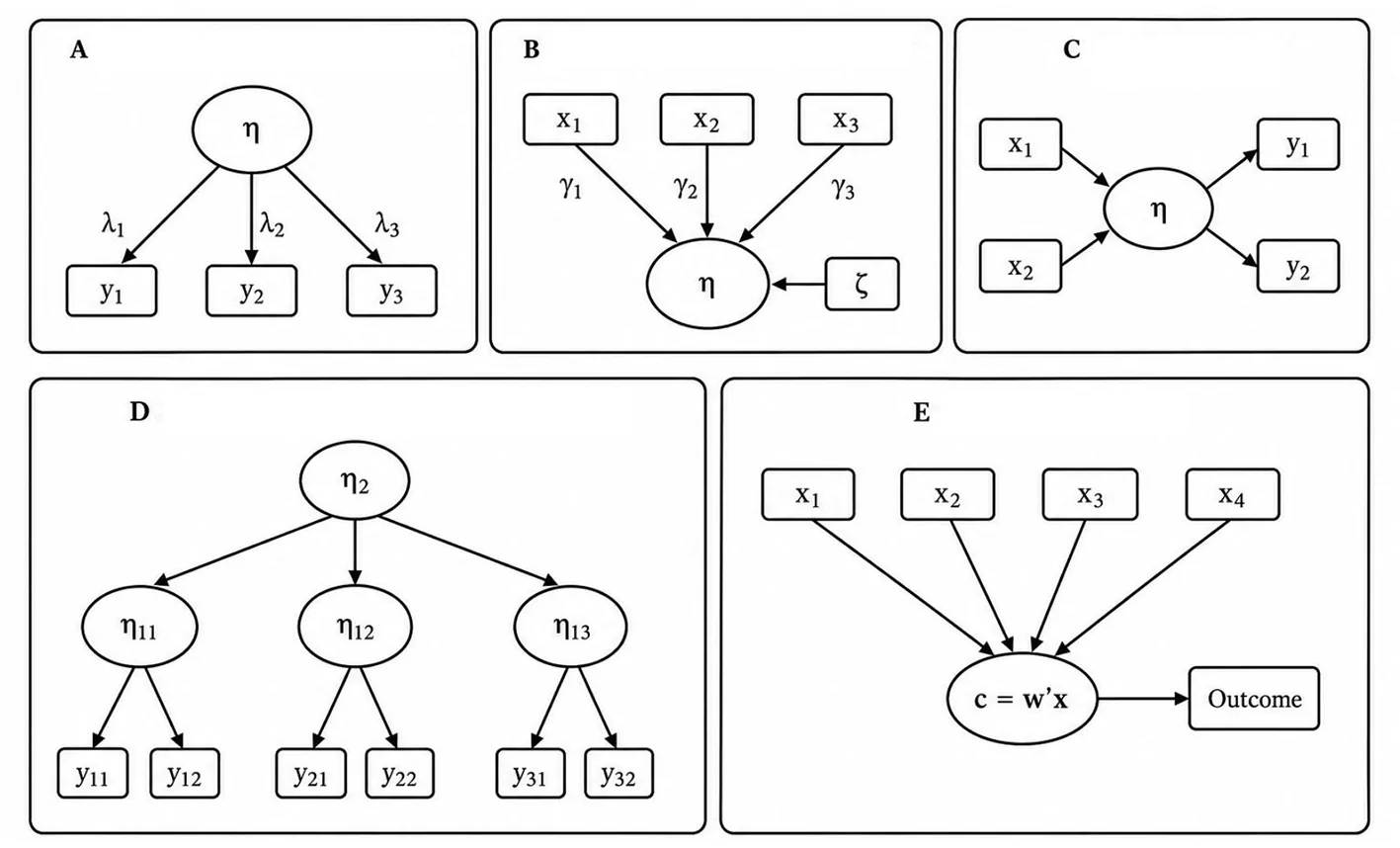
Schematic measurement-model families. Rectangles denote observed variables, ellipses denote theoretical or constructed variables, and residual terms represent omitted influences under the stated model. The diagrams are simplified to emphasize direction and model family. **(A)** Reflective common-factor model. **(B)** Causal-indicator/formative model. **(C)** MIMIC model. **(D)** Higher-order reflective-reflective model. **(E)** Composite model.

### 4.1 Reflective common-factor model

A reflective model posits that a latent variable accounts for covariation among effect indicators. In scalar form, each indicator is modeled as a loading times the factor plus an indicator-specific error. In matrix form:


$$\begin{array}{l} y=\Lambda \eta +\varepsilon \end{array}$$ (1)

The model implies that changes in η produce changes in y, conditional on the loading structure and local-independence assumptions. Reliability, convergent validity, discriminant validity, residual inspection, and overall model fit are relevant because the common factor is expected to reproduce patterned covariation. (Brown, 2015, p. 287–332) discusses higher-order CFA, reliability, and formative indicators within a broader CFA framework, while (Kline, 2023, p. 229–260) emphasizes specification, equivalent CFA models, and identification restrictions.

Reflective modeling is defensible only when the indicators can plausibly be interpreted as effects of the same underlying variable. Acceptable fit does not establish that interpretation. A network of direct indicator interactions, an omitted multidimensional structure, or common-method effects may yield similar covariance patterns. Therefore, factor analysis evaluates implications of a reflective theory; it does not manufacture the theory.

### 4.2 Causal-indicator (formative) model

A causal-indicator model specifies a theoretical variable as a function of observed causes and an omitted-cause disturbance:


$$\begin{array}{l} \eta =\gamma^{\prime }x+\zeta \end{array}$$ (2)

The disturbance ζ is substantively important. It distinguishes a causal-indicator model from a deterministic index and acknowledges that the listed indicators do not necessarily exhaust the causes of η (Diamantopoulos, 2006). Formative indicators need not be mutually correlated, and internal consistency is not a validity criterion. Relevant evidence concerns content coverage, indicator relevance, collinearity, external relations, sensitivity to indicator omission, and identification (Diamantopoulos et al., 2008).

The historical development predates later SEM treatments. Blalock (1964) discussed causal inference and indicator relations in nonexperimental research, and the sociological literature subsequently formalized models with observed causes and latent variables. Thus, causal-indicator reasoning has foundations that precede Bollen's later SEM treatments. Bollen's contribution was pivotal in clarifying and systematizing causal, composite, and effect indicators (Bollen, 2011), but formative logic should not be attributed to a single later source.

### 4.3 MIMIC models

The Multiple Indicators Multiple Causes (MIMIC) model integrates observed causes and reflective indicators around a latent variable. Hauser and Goldberger (1971) developed the treatment of unobservable variables in path analysis, and Jöreskog and Goldberger (1975) provided a general estimation model for a single latent variable with multiple indicators and multiple causes. Its two equations are:


$$\begin{array}{l} \eta =\gamma^{\prime }x+\zeta \end{array}$$ (3)

$$\begin{array}{l} y=\Lambda \eta +\varepsilon \end{array}$$ (4)

The MIMIC model is not merely a mixture of arrows. It separates the causal side, where x explains variation in η, from the measurement side, where y reflects η. Observed causes in a MIMIC model should not, however, be interpreted automatically as formative indicators; they may instead function as antecedents or covariates unless the construct theory assigns them a constitutive or causal-indicator role. This separation can support identification and external validation of a causal-indicator block. It also highlights why a formative block without consequences or reflective indicators may be empirically underdetermined. The model must still satisfy scaling, rank, and exclusion restrictions; adding arrows does not automatically identify it.

### 4.4 Higher-order measurement models

Higher-order models represent constructs at more than one level of abstraction. A reflective–reflective model posits a second-order factor that explains covariation among first-order factors, each of which explains its indicators:


$$\begin{array}{l} \eta 1=\Lambda_{2}\eta_{2}+\zeta 1 \end{array}$$ (5)

$$\begin{array}{l} y=\Lambda 1\eta 1+\varepsilon \end{array}$$ (6)

The higher-order relation can instead be constitutive. In a reflective–formative structure, reflective first-order dimensions jointly form a higher-order construct. Other combinations are possible when latent and emergent variables occupy different levels. (Henseler, 2021, p. 219–254) presents a six-type treatment of second-order constructs, while (Brown, 2015, p. 287–332) and (Kline, 2023, p. 255–260) discuss higher-order common-factor specifications and identification.

A recurring error is to infer higher-order logic from a diagram template or software option. The same visual arrangement can encode different theories (Lee and Cadogan, 2013). If technological, managerial, and learning capabilities are manifestations of a general capability, a reflective higher-order factor may be appropriate. If those dimensions are non-substitutable resources that constitute capability, a formative or composite higher-order model is more defensible.

### 4.5 Composite and component-based models

A composite is a weighted combination of observed variables or lower-order entities:


$$\begin{array}{l} c=w^{\prime }x \end{array}$$ (7)

Unlike a common factor, a composite does not explain indicator covariation through an underlying latent cause. Instead, the weighting rule determines the constructed variable; consequently, changing either the indicator set or the weights changes the meaning and numerical value of the composite (VanderWeele, 2022). Composite-based SEM provides model-based procedures for specifying, identifying, estimating, and assessing relationships among composites and latent variables. (Henseler, 2021, p. 38–84, 142–201) distinguishes composite, reflective, and causal-formative outer models and develops confirmatory composite analysis. Schuberth (2023) formalizes the Henseler–Ogasawara specification, while Henseler and Schuberth (2025) argue that factor-based and composite-based SEM should be treated as complementary rather than competing methodological traditions.

The treatment of measurement error also requires precise terminology. Observed components may contain unreliability even when a composite is algebraically exact conditional on its indicators and weights. Treating a composite score as error-free may attenuate or distort its structural relationships. Conversely, adding a reflective residual to every composite without substantive justification may unintentionally transform an emergent variable into a common-factor model. The proposed framework therefore asks where uncertainty arises—within the observed components, the weighting process, omitted components, or the structural relationship—rather than relying solely on whether the estimation software reports an explicit error term. Accordingly, algebraic exactness of the composite construction should not be conflated with error-free measurement of its observed components or with error-free use of the resulting score in a structural model.

These distinctions demonstrate that the principal model families differ not only in arrow direction or estimation procedure, but also in their ontological assumptions, treatment of indicator covariation, interpretation of residuals, and consequences of indicator omission. Table 2 summarizes these formal and interpretive contrasts among reflective common-factor, causal-indicator, MIMIC, higher-order, and composite specifications.

**Table 2:** Formal and interpretive contrasts among principal model families.

Model family	Core equation	Construct status	Primary validation emphasis	Characteristic risk
Reflective common factor	y = Λη + ε	Latent common cause of indicators	Loadings, residuals, reliability, convergent/discriminant validity, fit	Mistaking covariance reproduction for proof of ontology
Causal-indicator/formative	η = γ′x + ζ	Theoretical variable influenced by observed causes	Content coverage, relevance, collinearity, external relations, identification	Omitting disturbance or deleting domain-essential causes
MIMIC	η = γ′x + ζ; y = Λη + ε	Latent variable with observed causes and effects	Identification of both blocks, differential effects, fit, residuals	Treating causes and manifestations as equivalent indicators
Higher-order factor	η1 = Λ_2_η_2_ + ζ1; y = Λ1η1 + ε	Latent variable explaining lower-order factors	Level-specific loadings, residuals, scaling, alternative models	Confusing manifestation across levels with constitution
Composite	c = w′x	Constructed or emergent variable	Weighting rationale, component quality, nomological relations, model fit where available	Treating the score as a latent common cause or as error-free

Equations are schematic. A complete model must state scale setting, covariance restrictions, structural relations, residual assumptions^**^, and, where applicable, the explicit composite construction rule^**^.

## 5 Convergence, divergence, and unresolved methodological tensions

### 5.1 Areas of convergence

Across traditions, there is broad agreement on several points. Construct meaning should precede estimation; indicator roles should be explicit; different model families require different evidence; and *post hoc* respecification must be theoretically constrained. There is also agreement that a construct label does not fix a model and that higher-order structures require level-specific justification. These points establish a shared minimum standard for transparent measurement practice. Importantly, this convergence concerns the need for theoretically explicit specification rather than agreement on a single preferred measurement ontology or estimation tradition.

### 5.2 Areas of divergence

Disagreement persists regarding the ontological status of causal-indicator/formative specifications and composites, the interpretation of measurement error, and the criteria used to evaluate model adequacy. Common-factor approaches prioritize the reproduction of observed covariance through latent common causes. Causal-indicator approaches focus on observed antecedents that generate variation in a theoretically distinct variable. Composite approaches, by contrast, model constructed or emergent variables directly and may be estimated using procedures associated with either variance-based or covariance-based SEM. The underlying controversy is therefore not adequately represented as a simple opposition between CB-SEM and PLS-SEM. It concerns the type of construct being modeled, the role assigned to its indicators, and the empirical implications implied by that specification. Consequently, disagreements that appear to concern estimation methods may actually originate earlier, at the level of construct ontology and indicator interpretation.

A related tension concerns whether formative specification constitutes measurement in the conventional sense. Edwards (2011) argues that formative models may conflate a construct with its antecedents, whereas Bollen and Diamantopoulos (2017) maintain that causal-formative indicators are coherent when the theoretical variable is distinguished clearly from its observed causes. The alignment framework treats this disagreement as a diagnostic problem. When the specified indicators merely constitute a weighted list used to construct a score, the appropriate representation may be a composite or index. When they are theorized as causes of a distinct variable and omitted causes are represented by a disturbance term, a causal-indicator model is defensible. When neither distinction can be articulated, the specification remains conceptually unstable (Berbekova et al., 2025). The disagreement therefore cannot be resolved merely by choosing one terminology over another; it requires researchers to state whether the target variable exists theoretically beyond the selected indicators, how omitted causes are represented, and what empirical evidence could distinguish the proposed interpretation from a constructed score.

A second area of divergence concerns measurement error. In a common-factor model, indicator-specific error is explicitly separated from the latent common variable. In a causal-indicator model, the disturbance represents omitted causes of the theoretical variable, while the observed causal indicators themselves may also contain measurement error. In a composite model, the composite may be algebraically exact conditional on its components and weights even though the components or the composite's subsequent structural use remain subject to uncertainty. Thus, the question is not simply whether a model “contains an error term,” but where uncertainty is located and what the model treats as exact.

Higher-order constructs create a related ambiguity. A higher-order variable may represent a common factor manifested through lower-order dimensions, a theoretical variable influenced by those dimensions, or an emergent composite constituted by them. These specifications can appear visually similar while implying different relations among levels. Higher-order modeling therefore requires the ontology and indicator role to be justified separately at each level rather than inferred from a software template or hierarchical diagram.

These debates reveal both areas of agreement and unresolved differences among the principal measurement traditions. Table 3 summarizes their convergence, divergence, and continuing methodological tensions across construct status, indicator role, measurement error, validation criteria, and model interpretation. The comparison is intended to identify the assumptions that must be adjudicated theoretically and empirically rather than to designate one tradition as universally preferable.

**Table 3:** Convergence, divergence, and unresolved methodological tensions.

Issue	Common-factor perspective	Causal-indicator/formative perspective	Composite perspective	Diagnostic question
Ontology	Latent common cause	Theoretical variable affected by observed causes	Constructed or emergent variable	Does the construct exist independently of the indicator set and weighting rule?
Indicator omission	May reduce reliability without redefining the factor	May omit a cause and alter interpretation	Directly changes the composite	Would omission change the domain, only precision, or the score definition?
Error	Indicator-specific error and factor disturbance	Omitted causes represented by ζ; indicators may also be measured with error	Error may reside in components, their measurement, weighting, or structural use of the score	Where does uncertainty enter, and what is treated as exact?
Validation	Covariance fit, residuals, reliability, validity	Content, relevance, external relations, identification	Weighting rationale, component quality, model fit and nomological behavior	Which evidence could count against the stated interpretation?
Model choice	Theory plus covariance implications	Theory plus identified causal restrictions	Theory plus explicit construction and model assessment	Is the decision based on construct meaning or only empirical convenience?
Higher-order relation	General factor manifests in dimensions	Dimensions causally influence a higher theoretical variable	Dimensions constitute an emergent higher-order variable	Are lower-order dimensions effects, causes, or components?

The columns summarize ideal types. Hybrid models can combine roles, but each relation must retain a distinct interpretation. The same observed variables may therefore support different specifications only when the corresponding construct assumptions and evidential requirements are made explicit.

### 5.3 Why empirical fit cannot adjudicate ontology

Fit statistics evaluate the consequences of a specified model relative to data; they do not independently identify the nature of a construct. Competing models can be observationally equivalent, and near-equivalent models can fit almost equally well while making different causal claims. In addition, an overparameterized or weakly identified model may fit because it imposes few informative restrictions. Conversely, poorer fit does not by itself establish that the underlying construct theory is conceptually wrong; it identifies a discrepancy between the model's implications and the observed data that must be located and interpreted.

Therefore, the evidential sequence should be: define the construct, specify the indicator relation, establish identification, evaluate model implications, compare defensible alternatives, and interpret within context. This ordering prevents empirical fit from being used retrospectively to define construct meaning and makes explicit which assumptions are theoretical, which are statistical, and which remain underdetermined by the available data.

## 6 The construct–measurement alignment framework

### 6.1 Five-stage diagnostic process

The revised Construct–Measurement Alignment Framework is an iterative five-stage diagnostic process. It is not a mechanical decision rule that assigns a measurement model solely from a construct label. Rather, it requires a traceable sequence of reasoning from the ontological status of the construct to the role of its indicators, formal model specification, appropriate validation evidence, and the scope of substantive interpretation. Each stage constrains the decisions made at subsequent stages, while evidence of inconsistency at a later stage may require reassessment of earlier assumptions. Figure 2 presents this sequence and identifies the primary form of construct–measurement misalignment most directly associated with failure at each stage. Additional failures that operate across multiple stages are developed in the taxonomy presented in Table 4.

**Figure 2:**
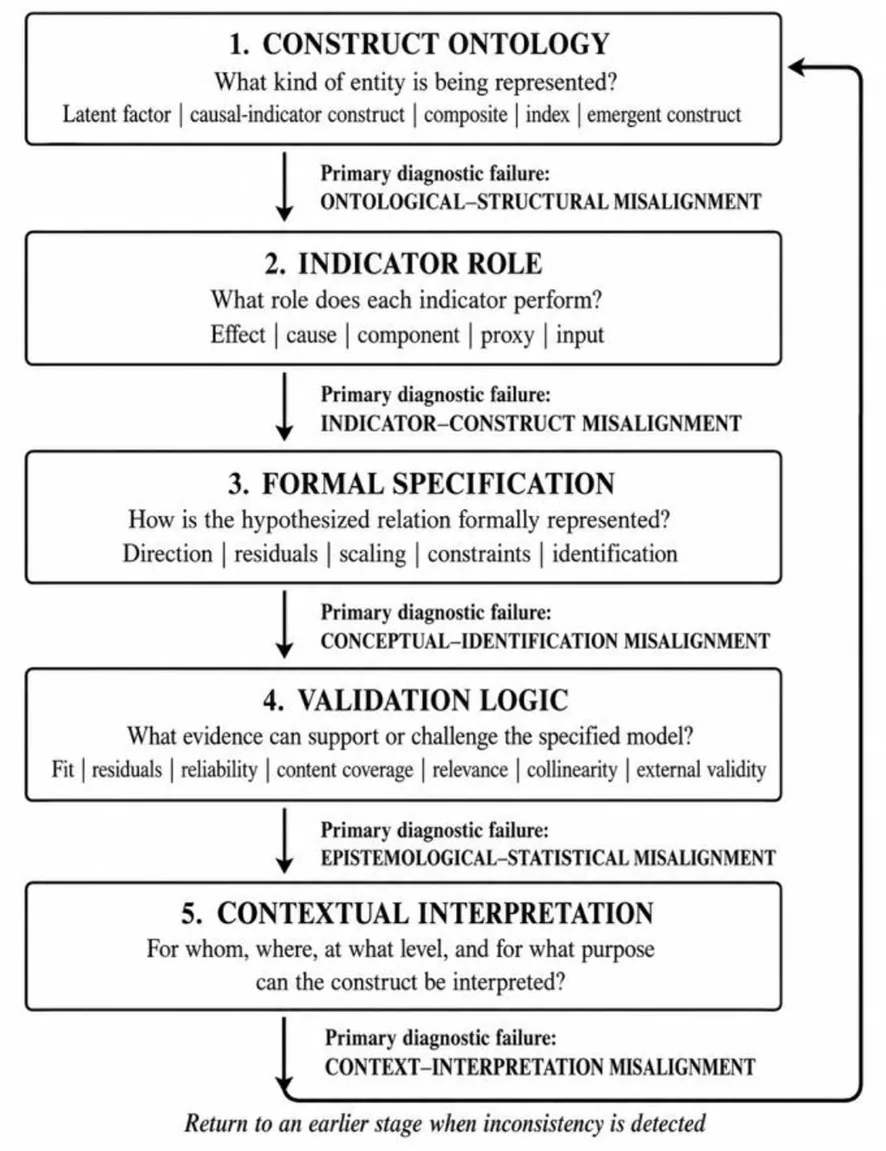
Five-stage Construct–Measurement Alignment Framework and diagnostic failure points. Each stage identifies the most immediate point at which construct–measurement reasoning can become inconsistent. The process is iterative rather than strictly linear; identification problems, inappropriate validation evidence, or incoherent substantive interpretation may require reassessment of the construct ontology, indicator roles, or formal specification. Error–specification and level-of-abstraction misalignments may operate across more than one stage and are therefore treated as cross-stage failures in Table 5.6.2 Stage 1: Construct ontology.

**Table 4:** Taxonomy of construct–measurement misalignment.

Misalignment	Defining inconsistency	Likely consequence	Primary corrective test
Ontological–structural	A construct conceptualized as a composite, index, or causal-indicator construct is represented as a reflective common factor, or vice versa.	Arrow direction, parameter meaning, and construct interpretation become inconsistent with the construct definition.	State what is expected to remain invariant when an indicator is removed, replaced, or reweighted.
Indicator–construct	Indicators are treated as interchangeable effects when they are non-substitutable causes or components, or treated as constitutive inputs when they are manifestations of a common construct.	Indicator deletion may produce construct drift, incomplete domain coverage, or unnecessary proliferation of dimensions.	Conduct omission, substitution, interchangeability, and domain-coverage analysis.
Epistemological–statistical	Construct status is inferred primarily from correlations, loadings, model fit, or software output.	Statistical behavior is mistaken for evidence about construct ontology, directionality, or causality.	Specify competing theoretical interpretations before using empirical fit or predictive performance to evaluate them.
Conceptual–identification	A conceptually meaningful model lacks sufficient scale-setting, rank, exclusion, outcome, or indicator restrictions for unique estimation.	Parameters are not uniquely estimable or become dependent on arbitrary constraints.	Establish model identification before interpreting parameter estimates.
Error–specification	The model locates uncertainty in the wrong part of the system or treats observed components or constructed scores as effectively error-free.	Structural relations may be attenuated or inflated and uncertainty may be understated.	Distinguish measurement error, component unreliability, omitted causes, and structural disturbances explicitly.
Level-of-abstraction	Relations among first-order and higher-order constructs are specified without clarifying whether lower-order dimensions are manifestations or constituents of the higher-order construct.	The same diagram may be interpreted differently across levels, producing inconsistent parameter meanings.	Specify construct ontology and indicator role separately at each level of abstraction.
Context–interpretation	A measurement specification is generalized across levels, populations, cultures, or decision purposes without demonstrating preservation of construct meaning.	Apparent comparability may mask substantively non-equivalent constructs or inappropriate generalization.	Evaluate invariance or sensitivity where appropriate, or explicitly delimit the scope of the construct definition and interpretation.

Several forms of misalignment may occur simultaneously. The taxonomy is diagnostic rather than mutually exclusive, and a failure identified at one stage may require reconsideration of decisions made at earlier stages.

### 6.2 Stage 1: construct ontology

The first stage specifies what kind of entity the measurement model is intended to represent: a latent factor, a causal-indicator construct, an index, a composite, or an emergent system property. The question is not metaphysical in an abstract sense. Rather, it concerns which features of the construct are expected to remain conceptually stable when indicators, weights, or contexts change. A common factor is hypothesized to generate variation in its effect indicators and may therefore, in principle, be represented by alternative indicators drawn from the same conceptual domain. A composite or index, by contrast, is partly constituted by the components and weighting rules used to construct it. A causal-indicator construct remains conceptually distinct from its observed causes because the specified indicators need not exhaust all possible causes, and omitted causes, disturbances, and downstream consequences can be represented explicitly in the model.

### 6.3 Stage 2: indicator role

The second stage classifies each indicator according to the role it is hypothesized to perform: effect, cause, component, proxy, or scoring input. Mixed indicator roles are permissible, but they should not remain implicit. MIMIC models, for example, include causal indicators on one side of a latent variable and reflective indicators on the other. Higher-order models can likewise contain reflective relations at one level and constitutive relations at another. The framework therefore requires researchers to state the expected conceptual and empirical consequences of changing, omitting, or substituting each indicator.

### 6.4 Stage 3: formal specification

The third stage translates the conceptual relation into an explicit formal specification using equations and a corresponding path diagram. This stage includes the direction of relations, residual or disturbance terms, scale-setting rules, fixed and freely estimated parameters, covariance restrictions, identification conditions, and the connection between measurement and structural components of the model. A label such as “formative” is insufficient if it does not indicate whether the construct is represented as a causal-indicator variable with a disturbance term, an exact index, a weighted composite, or a higher-order construct constituted by lower-order dimensions.

### 6.5 Stage 4: validation logic

The fourth stage determines which forms of evidence are appropriate for evaluating the specified model. Reflective models require assessment of factor loadings, residuals, reliability, convergent and discriminant validity, model fit, and plausible alternative factor structures. Causal-indicator models require evidence concerning content coverage, indicator relevance, collinearity, external or nomological relations, omitted causes, and model identification. Composite models require a defensible rationale for component selection and weighting, assessment of the quality and relevance of the components, model-level evaluation where applicable, and sensitivity analyses involving alternative component sets or weighting schemes. Validation should specify what empirical evidence would challenge the proposed model rather than merely assemble statistics that appear to support it.

### 6.6 Stage 5: contextual interpretation

The final stage constrains the substantive claims that can legitimately be made from the estimated construct. The appropriate specification may depend on the level of analysis, target population, cultural or institutional context, decision purpose, and consequences of indicator omission. Contextual contingency, however, does not imply unrestricted model choice. Researchers should explain why the same construct label represents a different ontology, indicator role, or level of abstraction when applied in a different setting. Without such justification, contextual flexibility risks becoming *post hoc* rationalization.

Taken together, Sections 6.1–6.6 establish the conceptual and methodological logic of the five-stage framework. The framework is not a mechanical checklist but an iterative diagnostic sequence for examining whether construct ontology, indicator role, formal specification, validation logic, and contextual interpretation are mutually consistent. Decisions at each stage constrain those made at subsequent stages, whereas identification problems, inappropriate validation evidence, or incoherent substantive interpretation may require researchers to return to an earlier stage and reconsider the construct definition, indicator roles, or formal specification. Table 5 translates the five stages into operational diagnostic questions that researchers, reviewers, and editors can use before estimation, during model assessment, and when reporting the final specification.

**Table 5:** Operational diagnostic questions in the five-stage framework.

Stage	Required statement	Minimum diagnostic evidence	Decision to revisit if evidence is inadequate
1. Construct ontology	Type and conceptual boundary of the construct	Domain definition; consequences of indicator substitution, omission, or reweighting	Whether the target is a latent factor, causal-indicator construct, composite, index, or emergent construct
2. Indicator role	Role of each indicator as effect, cause, component, proxy, or scoring input	Omission and substitution analysis; temporal or causal rationale; domain coverage	Indicator classification and completeness
3. Formal specification	Equations, diagram, scaling rules, residuals, parameter restrictions, and identification conditions	Identification analysis; implied covariance structure or explicit construction rule	Model family, constraints, and potentially omitted relations
4. Validation logic	Evidence appropriate to the specified model family	Fit and residuals, reliability, content coverage, relevance, collinearity, external validity, and sensitivity analysis as applicable	Operationalization, weighting rules, restrictions, indicator set, or model family
5. Contextual interpretation	Level of analysis, population, purpose, and scope of interpretation	Sensitivity across contexts; measurement invariance where relevant or explicit justification of non-equivalence	Construct definition, level of abstraction, and generalization claims

The diagnostic process is iterative. Identification failure, inappropriate validation evidence, or incoherent substantive interpretation may require revision of the construct ontology, indicator roles, or formal specification.

## 7 A taxonomy of construct–measurement misalignment

The five-stage framework generates a taxonomy by identifying points at which construct ontology, indicator role, formal specification, validation logic, and contextual interpretation become inconsistent. The taxonomy represents a central theoretical synthesis of the article because it moves beyond recognizing measurement misspecification as a general problem to specifying where the reasoning process breaks down, why the inconsistency arises, and what conceptual or statistical consequences are likely to follow.

Seven recurrent forms of construct–measurement misalignment are distinguished. Five correspond directly to the principal diagnostic failures associated with the five stages of the framework: ontological–structural, indicator–construct, conceptual–identification, epistemological–statistical, and context–interpretation misalignment. Two additional forms—error–specification and level-of-abstraction misalignment—cut across multiple stages and become especially important when researchers locate uncertainty incorrectly or specify relations among multidimensional and higher-order constructs. The seven categories are therefore diagnostic rather than mutually exclusive.

Table 4 summarizes the defining inconsistency associated with each form of misalignment, its likely consequences for measurement and interpretation, and the primary diagnostic response required within the framework.

### 7.1 Construct drift as a cumulative consequence

Construct drift occurs when successive measurement or statistical decisions progressively alter the meaning or boundary of a construct without an explicit theoretical revision. For example, a researcher may begin with a broad causal-indicator domain, remove indicators because they exhibit weak statistical relationships, retain only highly correlated components, and subsequently report the resulting measure as a reliable reflective scale under the original construct label. The final variable may demonstrate satisfactory psychometric properties while representing a substantially narrower theoretical domain.

Within the Construct–Measurement Alignment Framework, construct drift is therefore treated not as a single specification error but as a cumulative consequence of misalignment across stages. It can emerge when changes in indicator composition are not accompanied by reconsideration of construct ontology, indicator role, formal specification, or validation logic. Tracking these decisions across stages makes visible whether statistical refinement has preserved the intended construct or silently transformed it into a different one.

### 7.2 Epistemic underdetermination

A measurement specification is epistemically underdetermined when more than one substantively different representation—such as a reflective common-factor, causal-indicator, or composite model—remains compatible with the available evidence. This condition is particularly likely when the study provides insufficient theoretical, temporal, experimental, or external restrictions to distinguish among competing interpretations.

Model fit alone cannot resolve such under determination. Similar or even observationally equivalent covariance implications may arise from models that assign different meanings to constructs, indicators, and directional relations. Consequently, selecting the numerically best-fitting model does not by itself establish the ontological or causal status of the construct.

The appropriate response is to seek evidence capable of discriminating among competing interpretations. Such evidence may include temporal ordering, experimental or quasi-experimental variation, design-based restrictions, theoretically justified exclusion restrictions, content-domain analysis, external or nomological relations, and sensitivity to alternative specifications. Within the framework, epistemic underdetermination therefore signals a need for additional discriminating evidence rather than mechanical model selection.

## 8 Identification and empirically equivalent models

### 8.1 Identification as a conceptual requirement

Identification concerns whether the population parameters of a specified model are uniquely determined by its observable implications and imposed restrictions. It should therefore be established before estimation and before substantive interpretation of parameter estimates or validity evidence. For latent-variable models, scale-setting constraints are required; the available observed information must be sufficient relative to the number and configuration of free parameters; and the pattern of fixed, free, and excluded paths must permit a unique solution. (Kline, 2023, p. 255–260) summarizes identification requirements for higher-order and complex CFA structures, while (Henseler, 2021, p. 73–84) discusses identification within composite-based SEM.

Causal-indicator structures require particular attention because the construct is not identified merely by drawing arrows from observed causes to an unobserved variable. If η is specified only as a function of incoming indicators x and has no reflective indicators, structural outcomes, or other theoretically justified external restrictions, the corresponding causal effects or weights may not be uniquely recoverable from the observed covariance structure. A MIMIC specification can provide additional information by linking the latent variable to reflective indicators, but identification still depends on appropriate scale-setting, rank conditions, and the overall configuration of the model.

Identification is therefore more than a computational prerequisite or software diagnostic. Within the Construct–Measurement Alignment Framework, it is a conceptual requirement linking theoretical specification to empirical recoverability. A theoretically meaningful distinction between a reflective factor, causal-indicator construct, higher-order construct, or composite has limited empirical content if the parameters required to represent that distinction cannot be uniquely identified.

### 8.2 Equivalent and near-equivalent models

Empirically equivalent models generate the same observable covariance implications despite assigning different directions, parameterizations, or substantive interpretations to relations among variables. Near-equivalent models do not imply exactly the same covariance structure but may fit sufficiently similarly that they are difficult to distinguish in finite samples. Consequently, acceptable fit for a specified model does not establish that its structural interpretation is uniquely supported by the data.

The classical problem of equivalent SEMs is discussed by (Williams, 2012, p. 247–260), while (Kline, 2023, p. 194–196) emphasizes the need to consider theoretically plausible alternative models rather than treating a well-fitting specification as uniquely confirmed. In the second edition of Hoyle's *Handbook of Structural Equation Modeling*, (Preacher and Yaremych, 2023, p. 206–222) extend this reasoning through a broader treatment of model selection, emphasizing that model comparison should be based on substantively meaningful alternatives rather than fit statistics considered in isolation.

Equivalent and near-equivalent models place an important epistemic limit on what can be inferred from cross-sectional covariance data. For example, satisfactory fit of a reflective measurement model does not by itself exclude alternative directional structures, omitted common causes, or substantively different indicator–construct relations that generate the same or very similar observable implications. The problem is therefore not merely statistical model competition; it concerns whether the available design and evidence can discriminate among competing theoretical representations.

Within the Construct–Measurement Alignment Framework, model equivalence is treated as a form of epistemic under determination. Researchers should identify plausible alternative specifications, explain which theoretical assumptions, temporal ordering, experimental design features, exclusion restrictions, or external evidence discriminate among them, and moderate causal language when the available data do not permit such discrimination. Failure to consider equivalent alternatives can therefore produce epistemological–statistical misalignment even when conventional goodness-of-fit criteria are satisfactory.

### 8.3 Implications for model comparison

Model comparison is informative only when the alternatives are substantively interpretable, appropriately identified, and sufficiently comparable with respect to the construct being represented. Differences in fit should not be interpreted mechanically as evidence that one construct ontology is correct and another is false. Better fit may reflect greater flexibility, additional parameters, sample-specific characteristics, or assumptions unrelated to the substantive distinction under investigation.

Conversely, poorer fit for a theoretically important alternative can provide useful diagnostic evidence when the source of misfit can be connected to a specific assumption about construct ontology, indicator role, residual structure, or structural relations. The purpose of model comparison is therefore not to protect a preferred specification or to select the numerically best model automatically. It is to determine which theoretical or measurement assumptions are contradicted by the available evidence and whether revising those assumptions changes the meaning of the construct itself.

Accordingly, the framework recommends a sequence in which researchers first establish construct meaning and indicator roles, then verify identification, specify theoretically plausible alternatives, evaluate their empirical implications, and finally determine whether observed differences justify a change in formal specification or merely constrain the strength of substantive interpretation.

## 9 Worked applications of the framework

The purpose of the following applications is not to prescribe a universally correct measurement model for commonly used constructs. Rather, the examples demonstrate how the same construct label can imply different measurement specifications when its ontology, indicator roles, level of analysis, intended use, and validation requirements differ. Each application therefore follows the diagnostic logic of the Construct–Measurement Alignment Framework rather than assigning a model on the basis of the construct name alone.

### 9.1 Socioeconomic status

Socioeconomic status (SES) illustrates how alternative construct ontologies can imply different measurement specifications. If SES is theorized as an underlying social position that gives rise to multiple observable manifestations, a reflective model may be defensible. Such a specification, however, requires a substantive account of why a common latent status should generate variation in indicators such as educational attainment, income, occupational position, or perceived social standing rather than assuming this relation from their empirical correlations alone.

Alternatively, SES may be defined as a resource index constructed from income, education, wealth, occupation, or related socioeconomic attributes. Under this interpretation, the observed variables are components of a constructed score rather than interchangeable manifestations of a latent common cause. Removing a component or changing its weight can therefore alter the substantive meaning of the resulting score. An explicit index or composite specification is consequently more transparent than a reflective factor model.

A third possibility arises when observed socioeconomic resources are theorized as causes of a distinct latent social position that, in turn, produces observable consequences such as perceived status, social opportunities, or status-related evaluations. Such a structure corresponds more closely to a MIMIC model because causal indicators and reflective manifestations occupy different positions relative to the latent variable. Identification and the substantive distinction between causes and effects must then be justified explicitly.

The framework therefore prevents the common shortcut of labeling SES as “formative” simply because its indicators are heterogeneous. Indicator heterogeneity alone does not determine measurement ontology. The relevant questions are whether SES exists conceptually apart from its observed components, whether omitted causes are theoretically possible, whether observable manifestations or consequences are available, and whether the intended output is a latent-variable estimate or a deliberately constructed score.

### 9.2 Digital capability

Digital capability illustrates the importance of level of analysis and construct boundary. At the individual level, digital capability may be conceptualized as a latent ability that gives rise to performance on tasks, behavioral demonstrations, or item responses. Under this ontology, appropriately designed indicators function as manifestations of an underlying capability, making a reflective measurement model plausible.

At the organizational level, however, digital capability may refer to an emergent configuration of technological infrastructure, employee competencies, data governance, leadership support, financial resources, and organizational routines. These dimensions need not be highly correlated, and omission of one dimension may alter the substantive domain being represented. Where organizational capability is constituted by such non-substitutable dimensions, a higher-order composite or a model with reflectively measured first-order dimensions and a constitutive higher-order relation may be more defensible.

A different specification follows if organizational digital capability is theorized as a higher-order latent attribute that causes variation in several first-order capabilities. In that case, a reflective–reflective higher-order model is appropriate, provided that the hypothesized common-cause relation can be theoretically defended and empirically identified.

This application demonstrates why a measurement specification should not be transferred mechanically across analytical levels. Individual digital capability and organizational digital capability may share the same label while representing different theoretical entities. Contextual contingency is therefore legitimate only when changes in construct ontology and indicator roles are stated explicitly and accompanied by corresponding changes in formal specification and validation logic.

### 9.3 Service quality

Service quality provides a further illustration of how intended interpretation and decision purpose affect measurement specification. One conception treats service quality as an overall evaluative state that gives rise to perceptions of reliability, responsiveness, assurance, empathy, tangibles, or other service characteristics. Under this interpretation, the observed responses are manifestations of an underlying evaluation, making a reflective factor model plausible if the assumed common-cause structure is theoretically justified.

Service quality can also be defined as a diagnostic profile composed of distinct service attributes used for managerial assessment, prioritization, or resource allocation. Under this interpretation, the dimensions are constitutive components rather than interchangeable effects of a single latent factor. A composite or explicitly constructed index may therefore provide a more coherent representation because changing the included dimensions or their weights changes the meaning of the resulting service-quality score (Collier and Bienstock, 2009).

A higher-order specification is appropriate when first-order service-quality dimensions are themselves measured by reflective indicators. The relation between those first-order dimensions and the higher-order construct must nevertheless be specified separately. A reflective higher-order relation implies that the higher-order service-quality construct generates variation in its dimensions, whereas a constitutive higher-order relation implies that the dimensions jointly form the broader construct. The presence of a hierarchical structure therefore does not, by itself, establish the direction of measurement relations across levels.

The framework consequently changes the question from “Is service quality reflective or formative?” to a more diagnostic question: which conception of service quality is being represented, for what purpose, at what level of analysis, and through which indicator roles? The answer determines whether factor structure and reliability, content coverage and weighting, higher-order model fit, or sensitivity to alternative specifications provides the appropriate validation evidence.

Collectively, the three applications demonstrate that the Construct–Measurement Alignment Framework does not assign a fixed measurement model to a construct label. Instead, it evaluates whether construct ontology, indicator role, level of analysis, intended use, formal specification, validation logic, and contextual interpretation are mutually coherent. Socioeconomic status, digital capability, and service quality can each support more than one defensible specification, but alternative specifications represent different theoretical claims rather than statistically interchangeable ways of measuring the same construct.

Table 6 summarizes the resulting diagnostic decisions by showing how alternative construct definitions and contexts lead to different measurement specifications, validation requirements, and limits on substantive interpretation.

**Table 6:** Worked decisions generated by the Construct–Measurement alignment framework.

Construct and context	Ontology and indicator role	Defensible specification	Critical validation evidence	Interpretive limit
SES as underlying social standing	Latent entity; observed variables interpreted as manifestations	Reflective factor model, if a common-cause theory is defensible	Factor structure, residuals, reliability where appropriate, alternative models, external validity	Model fit does not establish that socioeconomic resources are necessarily effects of latent status
SES as resource score	Constructed score; non-interchangeable socioeconomic components	Explicit index or composite	Content coverage, component quality, weighting rationale, sensitivity to alternative components and weights	Score meaning changes when components or weights change
SES as observed causes plus manifestations	Observed socioeconomic causes of a distinct latent position with reflective consequences	MIMIC model	Identification, separation of causal indicators and reflective manifestations, scaling, residual structure, model fit	Directional interpretation requires theoretical and design-based justification beyond arrow placement
Individual digital capability	Latent ability; task or item responses interpreted as manifestations	Reflective CFA/SEM	Indicator quality, loadings, residuals, reliability, invariance where relevant	Generalization is limited to the population and performance domain represented by the indicators
Organizational digital capability as capability bundle	Emergent construct constituted by non-substitutable dimensions	Higher-order composite or reflectively measured first-order dimensions with a constitutive higher-order relation	Domain coverage, component quality, weighting rationale, collinearity where relevant, nomological relations, sensitivity analysis	Should not be interpreted as equivalent to an individual-level latent digital skill
Organizational digital capability as common higher-order attribute	Higher-order latent entity; first-order capabilities interpreted as manifestations	Reflective–reflective higher-order model	First- and higher-order factor structure, identification, residuals, alternative hierarchical models	Requires a defensible theory that the higher-order construct generates variation in first-order capabilities
Service quality as global evaluation	Latent evaluative state; service-dimension responses interpreted as effects	Reflective or higher-order reflective model	Factor structure, residuals, discriminant validity, reliability, alternative models	Dimension scores represent manifestations and should not automatically be interpreted as constitutive managerial inputs
Service quality as diagnostic profile	Constructed service profile; dimensions function as constitutive components	Composite or explicit index	Content validity, component quality, weighting rationale, sensitivity to alternative dimensions and weights	Internal consistency is not the governing validation criterion, and score meaning depends on included dimensions

The examples are theoretical demonstrations of the framework rather than claims that any specification is universally correct for a given construct label. The defensibility of each model depends on explicit assumptions concerning construct ontology, indicator role, formal specification, identification, validation logic, and context.

## 10 Testable theoretical expectations

The Construct–Measurement Alignment Framework yields a set of testable expectations concerning the conditions under which measurement-model decisions are more likely to become conceptually or statistically misaligned. These expectations follow from the relationships among construct ontology, indicator role, formal specification, validation logic, and contextual interpretation developed in the preceding sections. They are not intended as prescriptive rules. Rather, they identify directional or conditional relationships that can be examined through systematic coding of published studies, simulation, experimental vignette designs, and comparative model analyses. Practical methodological and reporting recommendations are presented separately in Section 11.

### 10.1 Expectation 1

Measurement misspecification is more likely when formal model selection occurs before researchers explicitly specify the ontology of the construct and the roles assigned to its indicators.

### 10.2 Expectation 2

Construct drift is more likely as indicator-retention decisions become increasingly dependent on sample-specific statistical performance and increasingly detached from prespecified construct boundaries and domain coverage.

### 10.3 Expectation 3

Epistemic under determination is greater when reflective, causal-indicator, and composite specifications are compared only through within-sample fit or predictive performance without theoretical, temporal, design-based, or external restrictions capable of discriminating among competing interpretations.

### 10.4 Expectation 4

The consequences of construct–measurement misalignment for structural parameter estimates are greater when the affected construct occupies a consequential position in mediation, higher-order, or multi-group models because misspecification can propagate to parameters that depend on the measurement specification.

### 10.5 Expectation 5

Identification problems are more likely to be treated primarily as estimation or software problems when distinctions among latent common factors, causal-indicator variables, composites, and constructed indices have not been established before formal model specification.

### 10.6 Expectation 6

More explicit reporting of construct ontology, indicator roles, formal equations, identification restrictions, validation logic, and contextual scope is associated with greater reproducibility of measurement decisions and greater conceptual comparability across studies.

These expectations convert the framework into an empirically examinable research agenda. For example, published studies can be coded to determine whether early specification of construct ontology is associated with fewer inconsistencies between conceptual definitions and estimated models. Simulation studies can examine how different forms of measurement misspecification propagate through mediation or higher-order structures. Experimental vignette studies can test whether providing researchers with explicit ontology and indicator-role information changes model-selection decisions. Comparative methodological studies can likewise examine whether transparent reporting improves agreement among independent analysts attempting to reconstruct or evaluate the same measurement specification.

## 11 Methodological and reporting recommendations

### 11.1 For researchers

Researchers should prepare a construct-specification statement before model estimation. At minimum, this statement should define the construct and its boundaries, distinguish it from conceptually adjacent constructs, classify the role of each indicator, present the corresponding equation or path diagram, explain scale-setting and identification conditions, specify the evidence required for validation, and delimit the context within which the resulting construct can be interpreted. Alternative specifications should be considered when they are theoretically plausible rather than introduced only in response to inadequate empirical fit.

Indicator deletion should be accompanied by an explicit assessment of its consequences for construct meaning. For reflective indicators, researchers should explain why removing an indicator does not materially alter the domain represented by the latent variable. For causal indicators, components, or scoring inputs, researchers should determine whether omission removes a theoretically relevant cause, narrows construct coverage, or changes the definition of the resulting composite or index. Statistical significance, loading magnitude, or weight magnitude alone is therefore insufficient justification for indicator retention or deletion.

Researchers should also report the assumptions that make the preferred specification distinguishable from plausible alternatives. Where reflective, causal-indicator, composite, MIMIC, or higher-order representations are theoretically defensible, model choice should be justified through construct theory, indicator roles, identification conditions, external evidence, and intended interpretation rather than through fit statistics alone.

### 11.2 For reviewers and editors

Reviewers should assess whether a manuscript's conceptual definitions, verbal descriptions, equations, diagrams, validation procedures, and substantive interpretations refer to the same measurement model. A recurrent inconsistency occurs, for example, when dimensions are described as constituting or forming a construct but are subsequently evaluated exclusively with criteria appropriate to interchangeable reflective indicators. A related problem arises when a constructed composite score is interpreted as though it were a latent common cause of its components.

Review reports should distinguish conceptual specification concerns from estimation or implementation preferences. When questioning a measurement model, reviewers should identify the assumption at issue and, where possible, indicate what theoretical, design-based, or empirical evidence could discriminate among plausible alternatives. This approach avoids treating disagreement over software, estimator, or model fit as equivalent to disagreement over construct ontology.

Editors can strengthen measurement transparency by requiring authors to identify the assumed construct type, report measurement equations or diagrams where appropriate, justify indicator-retention and deletion decisions, explain identification conditions, and disclose whether theoretically plausible alternative specifications were considered. Sabol et al. (2023) document continuing problems in PLS-SEM specification and reporting, while Sabol and Winton (2026) emphasize the relationship between methodological currency, citation practice, and research rigor. These concerns reinforce the value of reporting standards that require methodological guidance to be operationalized in model specification rather than acknowledged only through citation.

Taken together, these responsibilities position researchers, reviewers, and editors as complementary safeguards of construct–measurement alignment. Their evaluations should examine consistency among construct definitions, indicator roles, formal specifications, identification conditions, validation procedures, and substantive interpretations. They should also determine whether alternative specifications have been considered where theoretically warranted and whether the evidential basis for the preferred model has been reported transparently. Table 7 operationalizes these responsibilities as a minimum construct–measurement reporting checklist for manuscript preparation, peer review, and editorial evaluation.

**Table 7:** Minimum construct–measurement reporting checklist.

Item	Required disclosure	Reviewer question
Construct definition	Domain, boundary, level of analysis, and distinction from neighboring constructs	Could the same indicators plausibly represent a different construct?
Indicator role	Classification of each indicator as an effect, cause, component, proxy, or scoring input	Is the claimed indicator role consistent with the consequences of omission, substitution, or addition?
Formal specification	Diagram or equations, residual terms, scale setting, fixed and free parameters, and relevant covariance restrictions	Do the verbal description, equations, and arrows represent the same theoretical model?
Identification	Conditions and restrictions required for unique parameter estimation	Can the focal parameters be uniquely recovered without substantively arbitrary constraints?
Validation logic	Evidence appropriate to the assumed model family	What empirical observation or pattern would count against the proposed specification?
Indicator decisions	Rationale for retention, deletion, substitution, or weighting and their consequences for construct meaning	Did statistical modification alter the construct domain or interpretation?
Alternative models	Theoretically plausible competing, equivalent, or near-equivalent specifications	What theoretical or empirical evidence favors the preferred interpretation over plausible alternatives?
Context and scope	Population, level of analysis, decision purpose, invariance assumptions, and limits to generalization	Does the interpretation extend beyond the context in which the construct was operationalized?
Software and estimator	Software, estimator, relevant settings, and model syntax where feasible	Can another analyst reconstruct and reproduce the formal specification?

The checklist is model-neutral. It does not privilege common-factor, causal-indicator, or composite approaches; rather, it requires the assumptions, identification conditions, validation logic, and interpretive scope of the selected model to be made explicit.

## 12 Gaps addressed and future research

### 12.1 Gaps addressed by the present study

The present article addresses four related conceptual gaps in the measurement-model literature. First, it integrates construct ontology, indicator role, formal specification, validation logic, and contextual interpretation within a single iterative diagnostic process rather than treating these decisions as separate methodological tasks. Second, it develops a taxonomy that links identifiable forms of construct–measurement misalignment to their conceptual sources, likely consequences, and corresponding diagnostic responses. Third, it extends the analysis beyond the conventional reflective–formative distinction by incorporating MIMIC, higher-order, causal-indicator, and composite specifications within the same reasoning framework. Fourth, it embeds identification and model equivalence within construct–measurement reasoning rather than treating them solely as technical issues arising after model specification.

These contributions do not resolve substantive disagreements about the nature of particular constructs, nor are they intended to establish a universally preferred measurement tradition. Their value lies in providing a common diagnostic language for identifying where competing specifications diverge and what evidence would be required to discriminate among them. For example, disagreements about formative measurement can be reformulated as questions about whether the target is a theoretical variable influenced by observed causes, a constructed composite or index, or a set of antecedents that has been assigned a construct label without an adequate measurement rationale. Making these distinctions explicit constitutes a conceptual contribution because each interpretation entails different formal specifications, identification conditions, validation requirements, and limits to substantive inference.

### 12.2 Remaining empirical agenda

The framework generates an empirical research agenda that can evaluate both its diagnostic categories and its proposed mechanisms. First, content-analysis studies can code published SEM research for the seven forms of construct–measurement misalignment and examine their associations with reporting quality, specification changes, replication outcomes, and the consistency of substantive interpretation. Such studies would also permit assessment of the reliability and discriminant clarity of the taxonomy itself.

Second, simulation studies can isolate specific forms of misalignment and evaluate how they propagate into factor loadings, component weights, structural coefficients, indirect effects, standard errors, model comparisons, and multi-group conclusions. Particular attention should be given to conditions under which conceptual misspecification produces apparently acceptable global fit or predictive performance despite biased or differently interpreted structural parameters.

Third, experimental vignette studies can test whether the five-stage diagnostic process improves agreement among researchers, reviewers, and editors when they evaluate competing measurement specifications. These designs could manipulate the availability and sequencing of information about construct ontology, indicator roles, equations, identification restrictions, and validation evidence.

Fourth, cross-disciplinary applications can assess whether the seven-category taxonomy remains adequate in research settings dominated by administrative indices, digital trace measures, dynamic indicators, multidimensional scores, or algorithmically constructed variables. Such applications may reveal additional forms of misalignment or show that some categories should be divided, combined, or made context-dependent.

Further methodological work is also required for SEM specifications that combine latent variables and composites, particularly when observed components contain measurement error, the composite is endogenous, weights are estimated jointly with structural relations, or alternative weighting schemes yield materially different substantive conclusions. Recent developments in composite-based and component-based SEM expand the available formal tools, but the availability of an estimator does not itself determine the appropriate construct representation. Their substantive use therefore remains conditional on an explicit theory of the construct, defensible component selection, appropriate treatment of error, and clearly stated interpretive boundaries (Schuberth, 2023; Henseler and Schuberth, 2025).

## 13 Discussion

### 13.1 Theoretical implications

The central theoretical implication of the framework is that measurement misspecification is better understood as cross-domain misalignment than as a binary labeling error. A researcher may correctly describe a model as formative yet remain misaligned if the indicators are actually components of a constructed index, if the target variable is not conceptually distinct from those components, if a theoretically required disturbance is omitted, or if the resulting parameters are not identified. Conversely, a reflective model may achieve satisfactory empirical fit while remaining conceptually misaligned when the construct is explicitly constituted by non-substitutable dimensions.

This perspective shifts attention from the terminology assigned to a model toward the consistency of the reasoning chain that supports it. Construct validity cannot be localized in a single coefficient, fit statistic, or reliability estimate. It depends on a sequence of linked claims: the construct has been coherently defined; the indicators have defensible roles; the formal model represents those roles; its parameters are identified; the selected validation evidence addresses the implications of that model; and the resulting interpretation remains within the context for which the construct was specified. Failure at one stage alters the meaning and evidential value of the stages that follow.

The seven-part taxonomy provides a vocabulary for locating such failures. It distinguishes, for example, an ontological–structural inconsistency from an identification problem, an inappropriate error specification, or an unjustified extension across levels or contexts. This distinction matters because different forms of misalignment require different corrective actions. Improving global fit cannot resolve an ontological inconsistency, just as adding reliability evidence cannot identify an underidentified causal-indicator model.

### 13.2 Methodological implications

The framework cautions against two symmetrical forms of methodological reductionism. The first is factor-model universalism, in which abstract constructs are assumed to be latent common causes merely because their indicators are correlated or because factor-analytic tools are available. The second is composite pragmatism without construct theory, in which heterogeneous indicators are combined primarily because the resulting score predicts an outcome or performs well empirically. Common-factor and composite approaches can each be appropriate, but neither is justified by software availability, empirical fit, or predictive performance alone.

The integration of path diagrams and formal equations is therefore substantive rather than merely presentational. Equations reveal whether the model contains a disturbance term, whether a constructed score is defined deterministically from its components, how scale is established, and whether first- and higher-order variables are linked reflectively or constitutively. Diagrams expose arrow direction, omitted relations, indicator roles, and level-of-abstraction assumptions. Reporting both makes it easier to detect situations in which verbal descriptions, statistical models, and substantive interpretations refer to different construct representations.

Identification and equivalent models further constrain model comparison. A theoretically meaningful specification cannot support inference when its focal parameters are not uniquely recoverable, and superior fit does not establish a preferred ontology when observationally equivalent or near-equivalent alternatives remain plausible. Model comparison should therefore be interpreted as evidence about specified assumptions rather than as an automated procedure for discovering what a construct “really is.”

### 13.3 Implications for reliability and transparency

Reliability should be evaluated in relation to what is being measured or constructed and where error is assumed to enter the model. For reflective common-factor models, internal-consistency and factor-based reliability estimates concern the coherence and precision of effect indicators as manifestations of a common latent variable. These criteria are not universal requirements for causal indicators, components, indices, or composites.

For constructed composites, the relevant questions concern the quality and reliability of the observed components, the defensibility and stability of the weighting rule, sensitivity to alternative component sets or weights, and the consequences of component-level measurement error for structural relations involving the composite. A high internal-consistency coefficient does not establish that components are reflective effects, while defining a composite exactly as a weighted combination of observed variables does not imply that its components are measured without error. The location and consequences of uncertainty must therefore be stated explicitly.

Transparency is equally important for cumulative knowledge. Two studies may employ the same construct label and report similar standardized coefficients while estimating substantively different objects. Conversely, studies may legitimately use different measurement specifications when the underlying constructs differ by level of analysis, decision purpose, population, or theoretical definition. Conceptual comparability therefore requires more than numerical similarity (Sterner et al., 2024). The framework supports cumulative research by requiring researchers to disclose these differences rather than obscuring them through common labels or standardized reporting conventions.

### 13.4 Limitations

The proposed framework remains a conceptual and diagnostic synthesis and has not yet undergone independent empirical validation. The seven-category taxonomy has not been evaluated through systematic coding studies, expert-consensus procedures, simulation experiments, or tests of inter-rater reliability. Some categories may overlap in practice, and particular cases may involve multiple forms of misalignment simultaneously.

The framework also does not exhaust the measurement problems that may arise in longitudinal, multilevel, Bayesian, network, dynamic, or machine-learning-based measurement settings. These approaches may introduce additional questions concerning temporal stability, cross-level construct meaning, prior specification, network dependence, algorithmic feature construction, and model updating that are not fully represented in the present taxonomy.

Finally, the worked applications are theoretical demonstrations designed to illustrate the diagnostic logic of the framework. They do not provide empirical evidence that one specification is superior for socioeconomic status, digital capability, service quality, or any other construct. These limitations establish the appropriate scope of the current contribution and define priorities for subsequent empirical evaluation and refinement.

## 14 Conclusion

Measurement should begin with construct meaning, but defensible measurement requires more than a conceptual definition. It requires alignment among construct ontology, indicator roles, formal specification, identification, validation logic, and contextual interpretation. Reflective common-factor, causal-indicator, MIMIC, higher-order, and composite models are therefore not interchangeable techniques competing for universal superiority. Each represents a different set of claims about what a construct is, how observed variables relate to it, where uncertainty enters the model, and what can legitimately be inferred from the resulting estimates.

The Construct–Measurement Alignment Framework contributes a five-stage iterative diagnostic process and a seven-part taxonomy of recurrent misalignment. Together, they provide a structured means of identifying where construct–measurement reasoning fails, why the inconsistency matters, and what theoretical or empirical evidence is required to reconsider the specification. The framework also establishes important limits on inference: empirical fit cannot determine construct ontology, correlation cannot establish indicator direction, reliability statistics cannot substitute for construct theory, and an estimated structural model cannot by itself resolve observationally equivalent interpretations.

The practical implication is a shift from model labeling to model justification. Researchers should define the construct and its boundaries, classify indicator roles, express the hypothesized relations through equations and diagrams, establish identification, apply validation criteria appropriate to the model family, consider theoretically plausible alternatives, and restrict substantive interpretation to the context supported by the design and operationalization. Reviewers and editors, in turn, should evaluate the coherence of this reasoning chain rather than relying primarily on familiar model labels, fit thresholds, or standardized reporting routines.

The framework does not advocate one SEM tradition over another. Its central requirement is more fundamental: a measurement decision should be theoretically coherent, formally explicit, empirically assessable, and transparently reported. Treating construct–measurement alignment in this way provides a basis for more reliable estimation, more defensible interpretation, and greater conceptual comparability across studies.
